# Multiple exposures to poultry barn air and lipopolysaccharide synergistically increase the pulmonary expression of TLR‐4 and IL‐1β

**DOI:** 10.1002/1348-9585.12094

**Published:** 2019-10-27

**Authors:** Gaganpreet Kaur, Ram Saran Sethi

**Affiliations:** ^1^ College of Animal Biotechnology Guru Angad Dev Veterinary and Animal Sciences University Ludhiana Punjab India; ^2^ Department of Animal Biotechnology College of Animal Biotechnology Guru Angad Dev Veterinary and Animal Sciences University Ludhiana Punjab India

**Keywords:** IL‐1β, LPS, lungs, poultry barn air, TLR4

## Abstract

**Objective:**

Poultry farm workers are exposed to barn air and suffer from various respiratory disorders. Due to frequent prevalence of endotoxin in the farm settings workers can get co‐exposed to barn air and endotoxin. The study was aimed to explore the pulmonary damage following long‐term multiple exposures to poultry barn air with or without endotoxin.

**Methods:**

We studied the pulmonary expression of Toll‐like receptor 4 (TLR4) and Interleukin‐1β (IL‐1β) by exposing Swiss albino mice to poultry barn air for 6 days (Monday‐Saturday) in a week for 5 and 10 weeks. At the end of exposure, animals were challenged with lipopolysaccharide (LPS) or normal saline solution @80 μg/mouse intranasally. Histopathology, bronchoalveolar lavage (BAL) fluid and blood analysis were used to characterize lung damage. mRNA and protein expression of TLR4 and IL‐1β were evaluated using quantitative polymerase chain reaction (qPCR) and immunohistochemistry, respectively.

**Results:**

Histopathology along with TLC and DLC of blood and BAL fluid revealed lung damage following multiple exposures and damage was severe in combination with LPS. Exposures altered mRNA and protein expression of TLR‐4 and IL‐1β and the expression was more marked following 30 days of exposure. Further LPS co‐challenge showed a synergistic effect on the expression of TLR4 and IL‐1β.

**Conclusions:**

The data suggest that long‐term exposures with or without LPS caused lung damage and altered the pulmonary expression of TLR4 and IL‐1β.

## 1 INTRODUCTION

Poultry farming has made outstanding progress in structure and operation in the last three decades by evolving from mere backyard endeavor to one of the most specialized businesses. The increasing demands for poultry products along with technological advances have revolutionized the role and the structure of poultry industry.^1^ Presently, the poultry industry is the most organized sector in animal agriculture and is worth Euro 14 500 million.^2^ This sector has provided employment to around millions of people. However, the poultry workers come across certain health problems, particularly respiratory disorders.^3^

Livestock workers specially engaged in poultry farming are occupationally exposed to many respiratory hazards and display higher rates of respiratory symptoms including chronic obstructive pulmonary disease (COPD), asthma, chronic bronchitis, allergic alveolitis, and organic dust toxic syndrome.^4^ A variety of environmental agents including poultry dust can cause lung inflammation when inhaled. We recently reviewed that occupational contaminants including livestock, gases as well as pesticides alter the pulmonary innate responses.^5^ Poultry dust originates commonly from poultry residues, molds, and feathers and is biologically active as it contains microorganisms that increase the risk of various respiratory diseases.^4^

Lipopolysaccharide (LPS), a major component of the outer membrane of Gram‐negative bacteria,^6^ is associated with the development and/or progression of many types of lung diseases characterized by chronic inflammatory conditions.^7^ Single LPS exposure is sufficient to rapidly recruit neutrophils to the lung and to produce pro‐inflammatory cytokines and chemokines.^8^ The recognition of LPS is modulated by soluble factors, such as LPS binding protein and surfactant proteins that are present in airway lining fluid and influence the presentation of LPS to membrane‐bound CD14.^9^ Binding of LPS to CD14 triggers intracellular signaling that is mediated by Toll‐like receptor 4 (TLR4) in association with a secreted cofactor, MD‐2.^9^, ^10^

Toll‐like receptor 4 is also activated by the endogenous molecules or “danger signals” released during tissue injury in addition to bacterial endotoxin.^11^ Hence, TLR4 is the prototypical sensor of infection or injury that orchestrates the innate response via a sequential activation of both cell surface and endocytic signaling pathways.^12^ Increased expression of TLR‐4 gene has been associated with lung dysfunction following exposure to barn air.^5^ Elevated TLR‐4 subsequently activates nuclear factor kappa‐light‐chain‐enhancer of activated B cells (NF‐κB), which in turn directs the expression of various chemokines and pro‐inflammatory cytokines including Interleukin‐1β (IL‐1β) and tumor necrosis factor/TNF‐α.^13^

Interleukin‐1β is a potent pro‐inflammatory cytokine that is crucial for host‐defense response to infection and injury.^14^ Interleukin‐1β, encoded by the IL‐1β gene, has been associated with chronic inflammation and plays an important role in lung inflammatory diseases including lung cancer. Elevated levels of IL‐1 proteins, in particular IL‐1β greatly enhance the intensity of the inflammatory response.^15^

Bacterial endotoxins have been linked to several respiratory dysfunctions. We have earlier reported that single and multiple exposures (6 and 24 days) to poultry barn air results lung injury and alters the number of Clara and mucus‐producing goblet cells which may underlie altered responsiveness to LPS challenge in both WT and TLR9^−/−^ mice.^16^ Single and multiple exposures to swine barn air also depict pulmonary hyper‐responsiveness.^5^ Since most of the farmers work in the farms for a longer period of their life and there remains a strong possibility of endotoxin co‐exposure, we hypothesized that long‐term exposures (30 and 60 days) to poultry barn air along with LPS will alter the pulmonary innate immune response in terms of expression of TLR4 and IL‐1β. Hence we tested our hypothesis in a mice model of occupational exposure.

## 2 SUBJECTS AND METHODS

The experiment protocols were approved by the Institutional Animal Ethics Committee (IAEC), Guru Angad Dev Veterinary and Animal Sciences University (GADVASU), Ludhiana. Thirty six healthy male Swiss albino mice aged 6‐8 weeks were purchased from a small animal colony, maintained by the Lala Lajpat Rai University of Veterinary and Animal Sciences, Hisar, Haryana. Mice were maintained under controlled conditions of 12 hours light and 12 hours dark cycle at small animal housing hall, GADVASU, Ludhiana. The animals were given synthetic pelleted diet and water ad libitum. These mice were acclimatized for 1 week prior to start of experiment.

**Figure 1:**
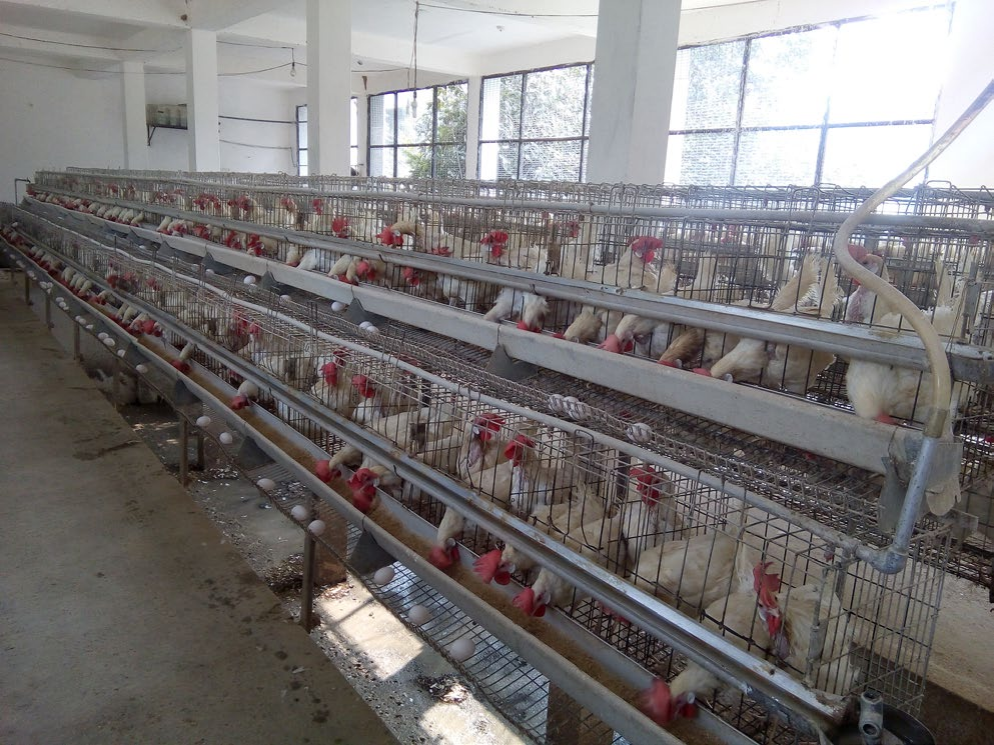
Layout of poultry house with cage housing system where animals were exposed to poultry barn air

Healthy mice (n = 36) were weighed and randomly divided into three groups viz. one control and two treatment (n = 12 each). The animals from the treated groups were transported to a poultry farm and were exposed to poultry barn air for 8 hours daily (9 am‐5 pm) and were brought back to the small animal house after exposure. Mice from treatment groups were kept in cages at height of 5 ft in the poultry farm with the cage housed system (Figure 1). Mice from Treatment groups 1 and 2 were exposed to poultry barn air for 5 and 10 weeks ie 6 days (Monday‐Saturday) in a week and a total of 30 and 60 days, respectively. The animals from the control group were transported daily to the poultry farm without exposing them to poultry barn air and were brought back to the small animal house to compensate the stress of transportation.

At the end of experiment, six animals from each of the three studied groups were administered *Escherichia coli* LPS @ 80 μL/mouse via intranasal route after anesthetizing with xylazine and ketamine combination anesthesia @ 1/10th of the body weight of mouse. The remaining animals were administered 80μl of normal saline solution (NSS) per mouse via the same route. The animals were euthanized with full dose of xylazine‐ketamine combination after 9 hours LPS/NSS challenge.

### 2.1 Collection of blood, BAL fluid and lung

Blood was collected via cardiac puncture using 1ml tuberculin syringe and stored in a vial containing Ethylenediamine tetraacetic acid (EDTA). The bronchoalveolar lavage (BAL) fluid was collected.^17^, ^18^ Briefly the left lung was ligated with cotton thread to prevent its flushing and 0.2 mL of phosphate buffer saline (1× Phosphate‐buffered saline [PBS]) was instilled into the in right lung through a catheter and aspirated. The process was repeated three times to collect 0.6 mL of BAL fluid. Blood and BAL fluid samples were processed for total leukocyte count (TLC) and differential leukocyte count (DLC) analysis on the same day. The right lung was chopped and placed in RNA later solution and stored at −80°C until further use for detection of mRNA expression of TLR4 and IL‐1β using real‐time PCR.^19^ Hence, the left lung was collected and fixed in paraformaldehyde solution at 4°C for 12 hours and was used for histopathological and immunohistochemical analysis.

### 2.2 TLC and DLC Analysis

Blood/BAL fluid (20 μL) was mixed with 380 μL of white blood cell diluting fluid for TLC analysis. Blood/BAL fluid smears were prepared and stained with Leishman stain and about 100 neutrophils or lymphocytes per sample were counted on each slide under the microscope at 40×. The cells are expressed as absolute number per mL of sample.

### 2.3 Histopathology

Lung samples fixed in paraformaldehyde were processed to obtain 5 μm thick paraffin sections on the Poli‐L‐Lysine coated slides with the help of a rotary microtome. The sections were stained with hematoxylin and eosin stain (H&E) for histopathological analysis. Morphologic changes (perivascular infiltration, peribronchial infiltration, sloughing of epithelium, size of perivascular space and thickening of alveolar septa) were graded semi quantitatively on H&E stained lung sections as described earlier.^20^ Each of these parameters was graded (0, normal/absent; 1, mild; 2, moderate; 3, severe) by an evaluator who was blinded to the identity of the samples.

### 2.4 Immunohistochemistry

Immunohistochemistry was carried out as described previously.^16^ Briefly, the sections were first deparaffinized, dehydrated, incubated with 3% H_2_O_2_ for 20 minutes to quench endogenous peroxidase and followed by boiling in tris borate EDTA and 1× PBS for antigen retrieval. The slides were incubated in a dark chamber with 1% Bovine serum albumin (BSA) and the sections were stained with primary antibodies against mice TLR4 (goat polyclonal TLR4; M‐16; sc12511; dilution 1:100) and IL‐1β (Anti IL‐1β rabbit affinity isolated antibody; dilution 1:25) followed by appropriate horseradish peroxidase‐conjugated secondary antibody (TLR4‐ Polyclonal rabbit anti goat; IL‐1β‐ Anti rabbit immunoglobulin G (IgG) produced in goat; dilution 1:100) to identify TLR4 and IL‐1β positive cells, respectively. The reaction was visualized using a color development kit (SK4100; Vector Laboratories). The sections were counterstained with methyl green. Controls for immunohistochemistry consisted of staining without primary antibody.

### 2.5 Real‐time PCR

The right lung from each animal stored in RNA later solution at −80°C was used for detection of mRNA of TLR4 and IL‐1β using real‐time PCR. The frozen lung tissue (100 mg) was used to extract total mRNA from all the samples using Trizol (Ambion; Life Technologies) method. The quality as well quantity of the resulting RNA was assessed spectrophotometrically using a Nanodrop (Thermo Fisher) and also by visualizing the ribosomal RNA bands via agarose gel electrophoresis. The concentration of total RNA varied between 1500‐3800 ng/μL in different samples. The amount of total RNA used for cDNA synthesis was adjusted to 400 ng/μL for each sample. Total RNA was reversed transcribed into cDNA using a High‐Capacity cDNA Reverse Transcription Kit (Catalog no. 4368814; Thermo Fischer Scientific) as per the manufacturer's instructions. Quantitative real‐time PCR was performed using SYBR green chemistry. Primer sequences used for amplification of TLR4 gene were 5′‐TGCTGAGTTTCTGATCCATGC‐3′ and 5′‐TGGCTAGGACTCTGATCATGG‐3′^21^ and for IL‐1β was 5′‐TGTAATGAAAGACGGCACACC‐3′ and 5′‐TCTTCTTTGGGTATTGCTTGG‐3′.^22^ The real‐time PCR reaction was carried out as duplicate and β‐actin was used as endogenous control. Data analysis for relative quantification was done using the ΔCT method.

### 2.6 Statistical analysis

Data from TLC, DLC and delta‐CT values are presented as mean ± SEM. Further, data were analyzed statistically using one‐way ANOVA followed by Duncan's post‐hoc test by using SPSS software. Results were considered statistically significant at *P* < .05.

## 3 RESULTS

Lipopolysaccharide challenge or 30 days multiple exposures to poultry barn air increased (*P* < .05) the TLC of blood along with significant leukocytosis, neutrophilia and lymphocytopenia as compared to the control group (Table 1). Multiple exposures of 60 days did not alter the TLC of blood, however caused increase (*P* < .05) in the neutrophil % and decrease (*P* < .05) in the lymphocyte % as compared to the control group. Lipopolysaccharide alone or multiple exposures (30 days) resulted in an increase (*P* < .05) in the TLC of BAL fluid along with neutrophilia (Table 1). The comparison between exposed groups revealed that 30 days of exposure resulted in a significant (*P* < .05) increase in TLC of blood and BAL fluid along with neutrophil % as compared to the group exposed for 60 days. Further, there was a significant (*P* < .05) increase in the TLC and neutrophil % of BAL fluid following 30 and 60 days of exposures in combination with LPS as compared to control, LPS alone or individual 30 or 60 days exposed group, respectively.

**Table 1:** TLC and DLC of blood and BAL fluid following multiple exposures to poultry barn air with or without LPS challenge

Group	TLC (per μL of blood)	Neutrophil (%) in blood	Lymphocyte (%) in blood	TLC (per μL of BAL)	Neutrophil (%) in BAL
Control	3741.67 ± 110.62^a^	30.83 ± 2.09^a^	69.17 ± 2.09^a^	83.33 ± 3.22^a^	24.33 ± 1.26^a^
LPS	10 308.33 ± 440.34^b^	41.83 ± 1.58^b^	58.17 ± 1.58^b^	161.16 ± 2.41^b^	31.83 ± 1.44^b^
30D	14 091.67 ± 540.43^c^	62.67 ± 2.33^c^	37.33 ± 2.33^c^	215.16 ± 7.66^c^	45.00 ± 2.08^c^
30D + LPS	17 568.83 ± 646.21^d^	73.83 ± 1.40^d^	26.17 ± 1.40^d^	556.67 ± 17.64^d^	49.33 ± 1.28^d^
60D	3175.00 ± 234.79^a^	38.50 ± 1.95^b^	61.50 ± 1.95^b^	118.17 ± 6.94^e^	27.83 ± 1.30^a^
60D + LPS	6833.33 ± 299.91^e^	62.83 ± 2.48^c^	37.17 ± 2.48^c^	334.33 ± 16.52^f^	36.50 ± 1.43^e^

Note

^a,b,c,d,e^No common superscript between two levels of an effect indicates significant difference (*P* < .05).

TLC and DLC are expressed as Mean ± SE.

Abbreviations: D, days exposed; DLC, differential leukocyte count; LPS, lipopolysaccharide; TLC, total leukocyte count.

### 3.1 Histopathology

**Figure 2:**
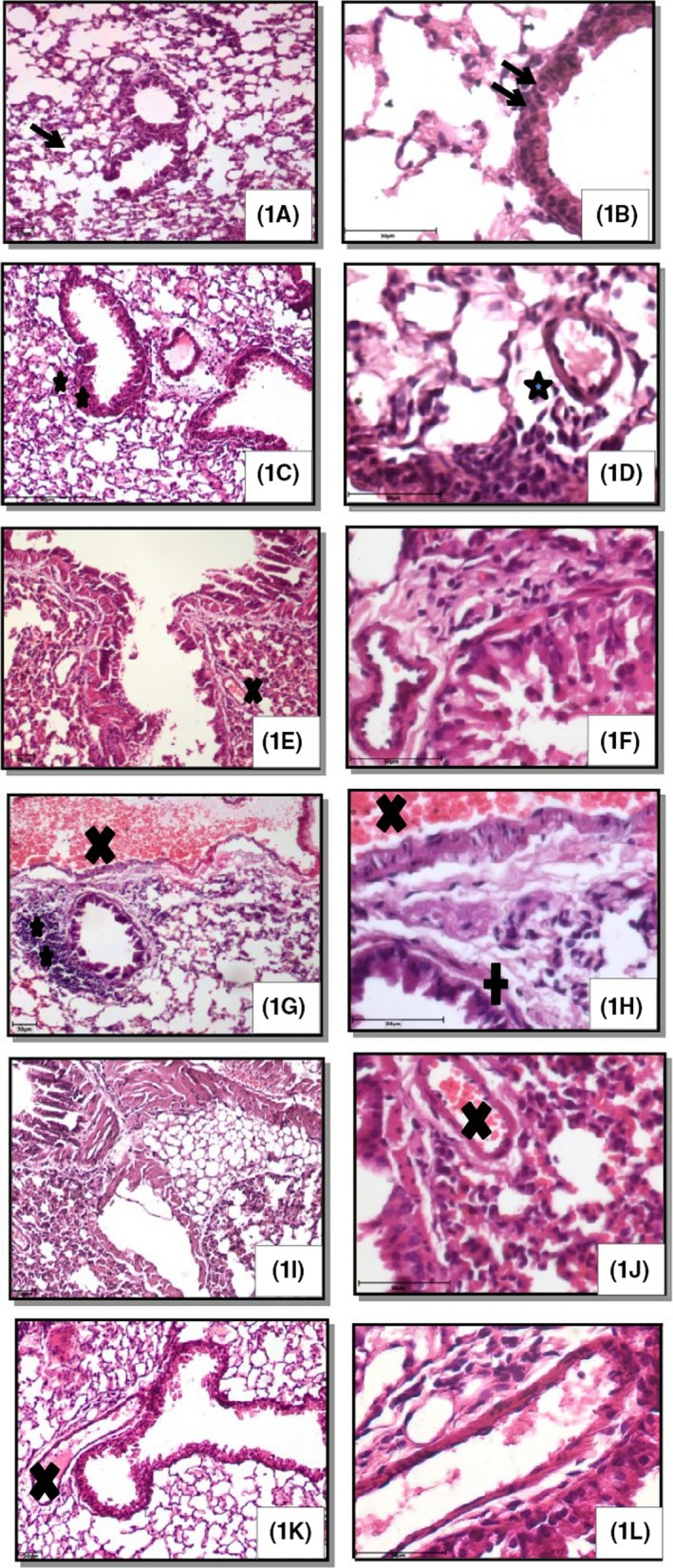
H&E staining: Lung sections of control mice (1A, B) showed normal alveolar septa (arrow) and bronchial epithelium (double arrows). Also seen perivascular (star) and peribronchial infiltration (double stars), congestion (X) following exposure to lipopolysaccharide (LPS) (1C, D), 30 days (1E, F), 60 days (1I, J), 30 days followed by LPS (1G, H) and 60 days followed by LPS (1K, L). Original magnification 1A, C, E, G, I, K: 100×; 1B, D, F, H, J, L: 400×. ^A,B,C,D,E,F^No common superscript between two levels of an effect indicates significant difference (*P* < .05)

Hematoxylin and eosin stained lung sections from the control group showed normal histo‐architecture of lungs (Table 2; Figure 2). However, LPS challenge revealed lung damage characterized by infiltration of mononuclear cells around perivascular and peribronchiolar regions, sloughing of epithelium and thickening of the alveolar septa (Table 2; Figure 2). Similarly multiple exposures of 30 and 60 days alone or in combination with LPS resulted in congestion, thickening of alveolar septa, peribronchial and perivascular infiltration indicating lung damage. Further, LPS challenge in combination with 30 and 60 days of multiple exposures resulted more lung damage (Table 2; Figure 2).

**Table 2:** Histopathological changes following multiple exposures to poultry barn air with and without LPS challenge

Inflammatory reaction	Perivascular infiltration	Peribronchial infiltration	Sloughing of epithelial surface	Size of perivascular space	Thickening of alveolar septa
Control	−/+	−/+	−	−	−
LPS	++	++	+	++	+
30D	++	++	+	++	++
30D + LPS	++/+++	+++	++	++	++
60D	++	+	+	++	+/++
60D + LPS	++	++	+	++	+/++

Abbreviations: −, normal; +, mild; ++, moderate; +++, extensive, D, days exposed; LPS, lipopolysaccharide.

### 3.2 Toll‐like receptor 4

**Figure 3:**
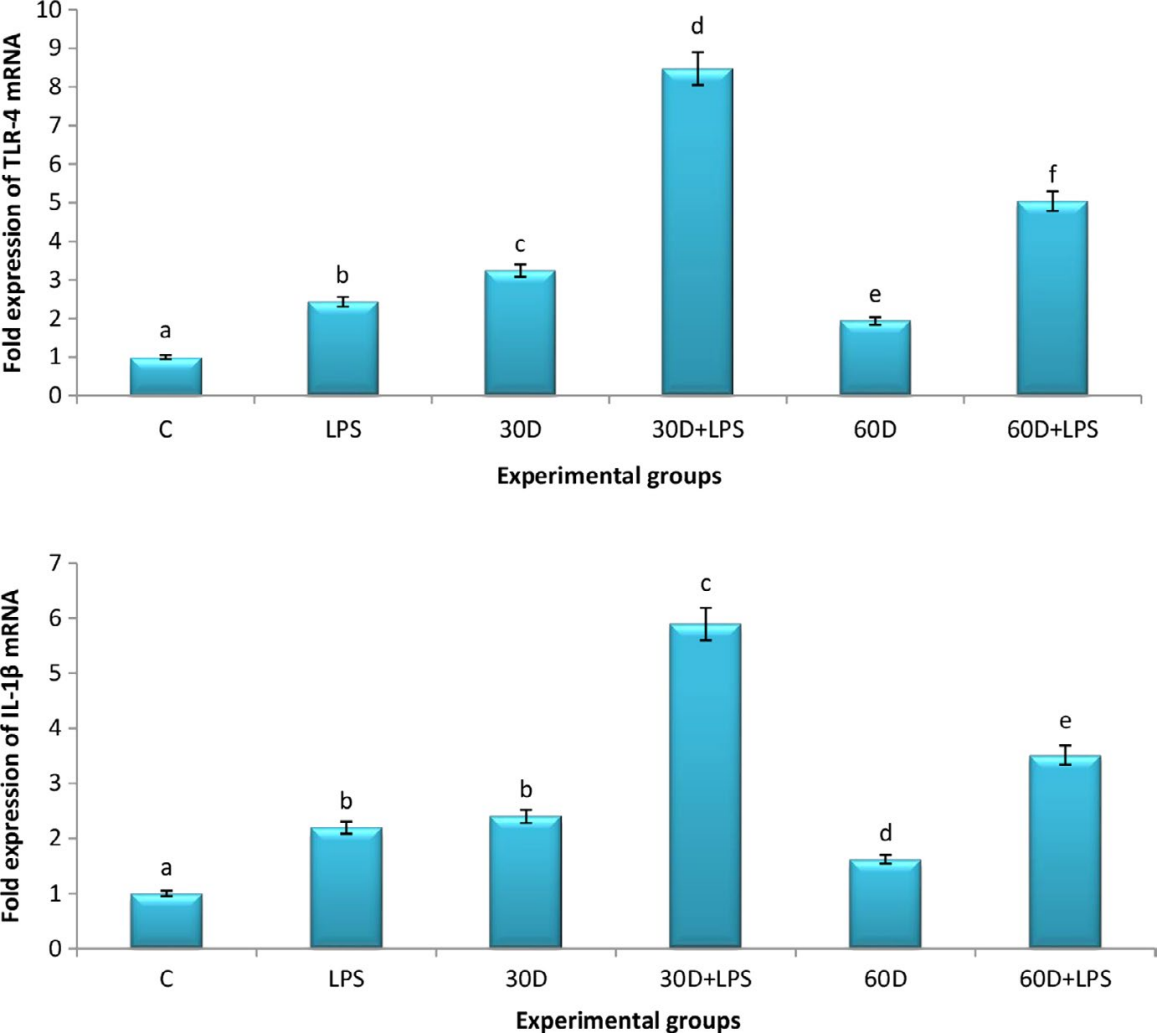
mRNA expression of Toll‐like receptor 4 (TLR4) and IL‐1β mRNA following multiple exposures to poultry barn air with or without LPS challenge. C, control; D, days exposed; LPS, lipopolysaccharide

Lipopolysaccharide, 30 and 60 days multiple exposures resulted increase (P < .05) of 2.43, 3.24 and 1.94 folds in the mRNA expression of TLR4 as compared to control group, respectively (Figure 3). The mRNA expression of TLR‐4 was higher following 30 days of exposure as compared to 60 days of exposure. Multiple exposures of 30 and 60 days in combination with LPS further increased (*P* < .05) the expression of TLR‐4 mRNA by 8.47 and 5.04 fold as compared to LPS alone or individual 30 or 60 days exposed group, respectively.

**Figure 4:**
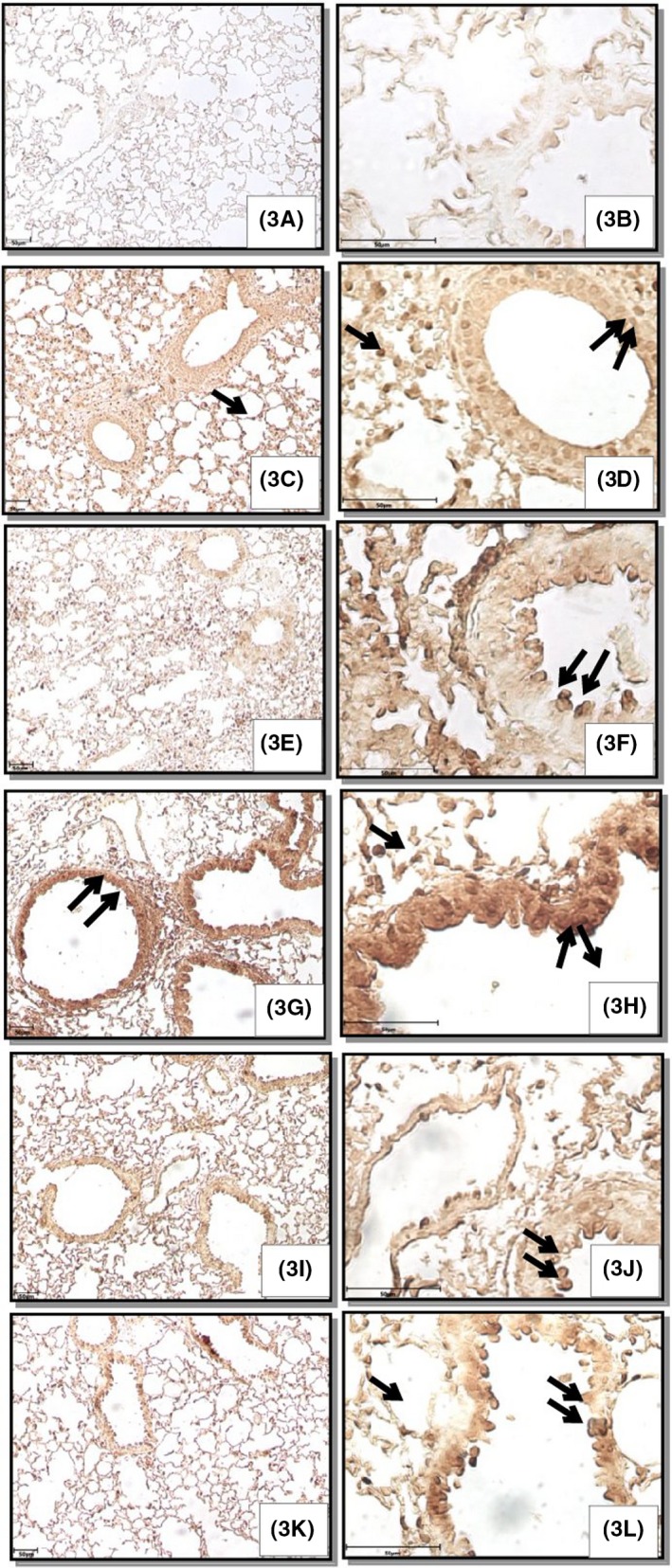
Lung sections showing Toll‐like receptor 4 immunopositive reactivity in airways epithelium (double arrows) and alveolar septal cells (arrow) in control (3A, B), LPS (3C, D) and multiple exposures of 30 days alone (3E, F) and in combination with LPS (3G, H), 60 days alone (3I, J) and in combination with LPS (3K, L). Immunohistochemistry (IHC), original magnification A, C, E, G, I, K: 100×; B, D, F, H, J, L: 400×

There was weak TLR4 immunopositive reactivity in the airways epithelium and alveolar septal cells in the lungs of control animals (Figure 4). Lipopolysaccharide challenge showed a mild TLR‐4 immunopositive reaction in airways epithelium and alveolar septal cells. Multiple exposures of 30 and 60 days resulted in moderate TLR‐4 immunopositive reaction in airways epithelium and alveolar septal cells. Further, 30 days of exposure along with LPS caused intense TLR‐4 immunopositive reactivity in airways epithelium and alveolar septal cells. However, the intensity of TLR‐4 immunopositive reaction was mild following 60 days of exposure in combination with LPS.

### 3.3 Interleukin‐1β

Lipopolysaccharide or multiple exposures of 30 and 60 days increased (*P* < .05) the expression of IL‐1β mRNA by 2.19, 2.4 and 1.62 fold compared to control, respectively (Figure 3). The mRNA expression of IL‐1β was significantly higher following 30 days of exposure as compared to 60 days of exposure. Lipopolysaccharide along with 30 and 60 days of multiple exposures further increased (*P* < .05) the expression of IL‐1β mRNA by 5.89 and 3.52 fold as compared to LPS alone or individual 30 or 60 days exposed group, respectively.

**Figure 5:**
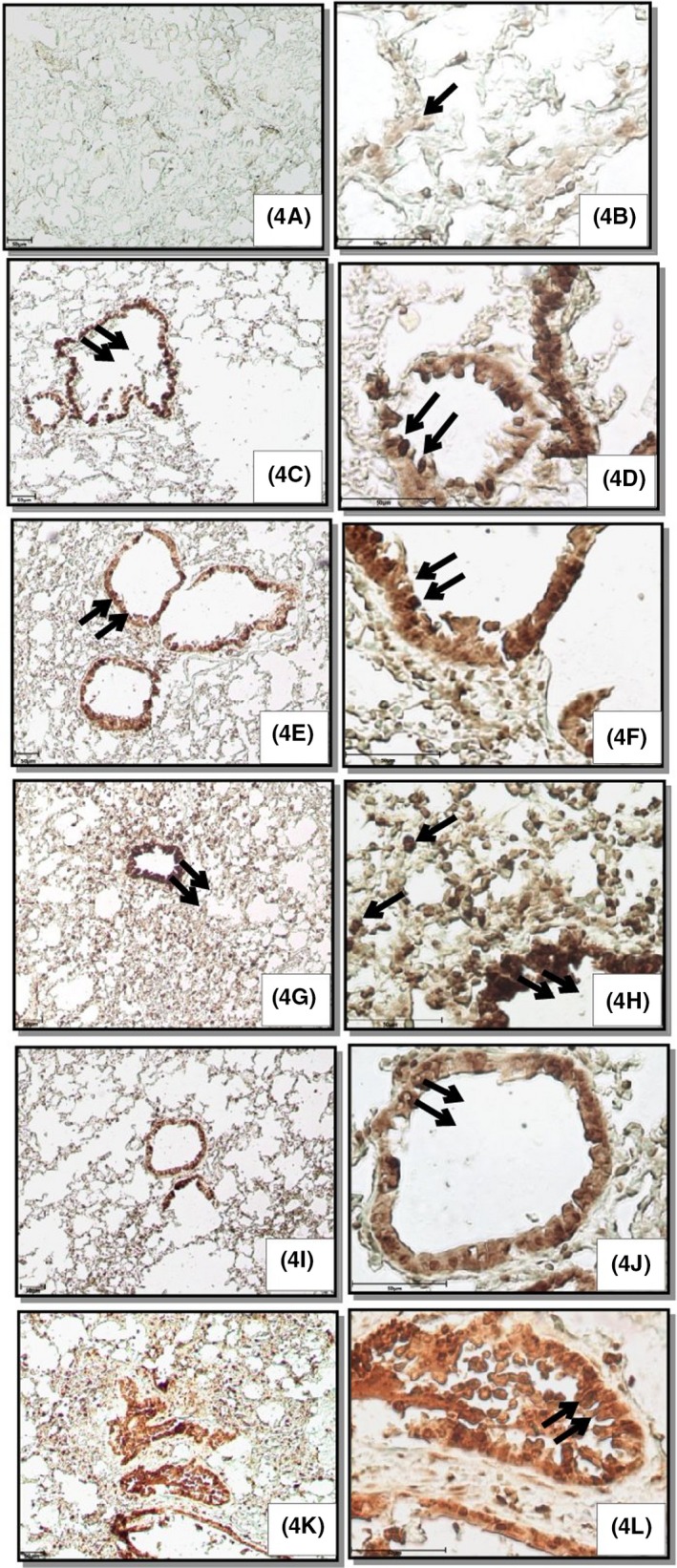
Lung sections showing immunopositive reactivity for IL‐1β in airways epithelium (double arrows) and alveolar septal cells (arrow) in control (4A, B), LPS (4C, D), multiple exposures of 30 days alone (4E, F) and in combination with LPS (4G, H) and 60 days alone (4I, J) and in combination with LPS (4K, L). Immunohistochemistry, original magnification A, C, E, G, I, K: 100×; B, D, F, H, J, L: 400×

Control group mice showed weak immunopositive reactivity of IL‐1β in alveolar septal cells and epithelium (Figure 5). Lipopolysaccharide or multiple exposures of 30 and 60 days showed moderate immunopositive IL‐1β reactivity in airways epithelium and alveolar septal cells (Figure 5). Further, 30 and 60 days of exposure in combination with LPS resulted in intense and strong immunopositive IL‐1β reactivity in airways epithelium, alveolar septa, and in peribronchial and perivascular infiltrating cells, respectively.

## 4 DISCUSSION

This study aimed to investigate the association between pulmonary expression of TLR4 and IL‐1β and long‐term multiple exposures (30 and 60 days) to poultry barn air with or without endotoxin challenge. While barn air exposure has been linked with various types of pulmonary dysfunction, there is no information available on the long‐term exposure alone or in combination with pathogen‐derived molecules such as LPS. We present first data on the pulmonary expression of TLR4 and IL‐1β following long‐term multiple exposures (up to 60 days) to poultry barn air in conjunction with endotoxin. The data suggest that endotoxin when combined with multiple exposures to poultry barn air alter the histomorphology of the lung and pulmonary expression of TLR‐4 and IL‐1β.

Blood findings are used to determine various systemic functions, health of animals under various environmental conditions and to diagnose hemolysis.^23^ Lipopolysaccharide challenge or 30 days of multiple exposures caused leukocytosis, neutrophilia and lymphocytopenia. Lipopolysaccharide alone or multiple exposures of 20 days to swine barn air have been reported to cause leukocytosis.^20^, ^24^ The damage to lymphocytes reflects immunotoxicity.^17^ The data taken together suggest that 30 days of exposure showed significant neutrophilia and lymphocytosis as compared to 60 days of exposures and these changes were more marked when exposures were combined with LPS.

We use histopathology and BAL fluid analysis to determine lung inflammation. Bronchoalveolar lavage fluid is a hallmark of lung inflammation^25^ as it provides inflammatory cell profiles in affected lung tissues to support the diagnosis of specific disorders^26^ and to detect the presence of respiratory pathogens.^27^ Lung inflammation is dominant by the infiltration of neutrophils, monocytes and lymphocytes in the lung.^28^ Lipopolysaccharide challenge increased the TLC of BAL fluid along with neutrophilia as observed earlier^20^ and these findings also validated our mouse model. Bronchoalveolar lavage fluid count of neutrophils is significantly higher during lung damage.^29^ Lipopolysaccharide inhalation induces neutrophilic airway inflammation resulting increase in neutrophil and total cells in the BAL fluid.^9^ Similarly multiple exposures of 30 days alone or in combination with LPS increased the TLC and neutrophil % of BAL fluid as compared to the control group and LPS or 30 days exposed group alone. The data taken together indicate that 30 days of exposures resulted in increased lung damage as compared to the 60 days exposed group which suggests that prolonged multiple exposures may induce adaptation. Further, damage was more marked when exposures were combined with LPS. Hence, LPS may be a confounding factor for lung damage when combined with multiple exposures to poultry barn air.

Multiple exposures (30 and 60 days) to poultry barn air exposure resulted in congestion, thickening of alveolar septa, peribronchial and perivascular infiltration indicating lung damage. Lung damage had been reported following exposure to poultry barn air^16^, ^30^ and swine barn air.^24^ Particles of respirable size remain airborne longer and penetrate deeper within the respiratory system resulting in lung injury.^31^ Lipopolysaccharide challenge in combination with 30 and 60 days of multiple exposures to poultry barn air resulted in increase in the congestion, sloughing of epithelial surface, peribronchial infiltration and perivascular infiltration. Endotoxins/LPS have been linked to lung damage in rat^32^ and mice^20^ and LPS exposure along with various environmental pollutants such as pesticides alter the pulmonary responses to the lung damage.^20^ The data indicate that multiple exposures of 30 and 60 days result lung damage and post administration of LPS further enhances the poultry barn air induced lung inflammation.

Lipopolysaccharide resulted in an increase in the mRNA expression of TLR4 and IL‐1β and immunopositive reaction in airways epithelium and alveolar septal cells. Toll‐like receptor 4 is normally expressed in the endothelial cells, airway epithelium, neutrophils, dendritic cells and macrophages of lungs.^33^ TLR4 is the key LPS receptor and LPS binding with TLR4 activates the TLR4 mediated inflammatory response.^34^ Multiple exposures (30 and 60 days) increased the mRNA expression and immunopositive reactivity of TLR4 and IL‐1β and the increase was more marked following 30 days of exposure as compared to 60 days. Toll‐like receptor 4 activation results in the expression of various pro‐inflammatory cytokines including IL‐1β and TNF‐α.^13^ Interleukin‐1β is an important cytokine that regulates the expression of several genes involved in various inflammatory processes.^35^ Endotoxin/LPS induces pulmonary expression of IL‐1β^36^ via activation of the NF‐κB pathway.^37^ Increased expression of IL‐1β has been associated with chronic inflammation and plays an important role in various lung inflammatory diseases including COPD or asthma^38^ and lung cancer.^39^ Hence the data suggest a TLR‐4‐dependent increase in the expression of IL‐1β following multiple exposures to poultry barn air.

Multiple exposures to poultry barn air in combination with LPS further increased the pulmonary expression of TLR‐4 and IL‐1β mRNA as compared to the LPS alone or individual exposure group without LPS, respectively. LPS induces systemic inflammation and is a major pathogenic element.^6^ Toll‐like receptor 4 is essential for LPS recognition and plays a role in the host defense against Gram‐negative bacteria, inflammation, injury and stress.^40^ Co‐exposure of LPS is known to alter pulmonary responses during exposure to various environmental pollutants.^5^, ^17^ Our design of experiment lacks the validation of results on human cell lines, however, the data suggest that 30 days multiple exposures with or without LPS altered histomorphology and expression of TLR‐4 and IL‐1β significantly as compared to 60 days exposures. Further multiple exposures in combination with LPS synergistically increased the expression of TLR‐4 and IL‐1β mRNA.

## 5 CONCLUSIONS

We conclude that multiple exposures of 30 and 60 days to poultry barn air resulted in lung damage and damage was more severe when combined with LPS. Exposures of 30 and 60 days significantly altered the mRNA and protein expression of TLR‐4 and IL‐1β. Expression of TLR4 and IL‐1β showed a synergistic effect when combined with LPS. Since livestock workers are always at risk to endotoxin exposures, the data indicate a comprehensive relook while treating respiratory illnesses in these workers.

## DISCLOSURE

*Approval of the research protocol*: The experiment protocols were approved by the Institutional Animal Ethics Committee (IAEC), Guru Angad Dev Veterinary and Animal Sciences University (GADVASU), Ludhiana. *Informed consent*: NA *Registry and the registration no. of the study/trial*: IAEC/2017/849‐80 dated 07/08/2017. *Animal studies*: The study involved the use of male Swiss albino mice (n = 36) aging 6‐8 weeks. *Conflict of interest*: The authors declare no conflict of interest.

## AUTHOR CONTRIBUTIONS

The research work is part of Master's thesis of GK. RSS is major supervisor of GK.
